# Green Preparation of Fluorescent Carbon Quantum Dots from Cyanobacteria for Biological Imaging

**DOI:** 10.3390/polym11040616

**Published:** 2019-04-03

**Authors:** Xi Wang, Pei Yang, Qian Feng, Taotao Meng, Jing Wei, Changyan Xu, Jingquan Han

**Affiliations:** College of Materials Science and Engineering, Nanjing Forestry University, Nanjing 210037, China; wxde1994@163.com (X.W.); ypde1990@163.com (P.Y.); njfu_fq@njfu.edu.cn (Q.F.); 15074897646@163.com (T.M.); weijing941017@163.com (J.W.)

**Keywords:** cyanobacteria, carbon quantum dots, hydrothermal method, bioimaging

## Abstract

Biomass-based carbon quantum dots (CQDs) have become a significant carbon materials by their virtues of being cost-effective, easy to fabricate and low in environmental impact. However, there are few reports regarding using cyanobacteria as a carbon source for the synthesis of fluorescent CQDs. In this study, the low-cost biomass of cyanobacteria was used as the sole carbon source to synthesize water-soluble CQDs by a simple hydrothermal method. The synthesized CQDs were mono-dispersed with an average diameter of 2.48 nm and exhibited excitation-dependent emission performance with a quantum yield of 9.24%. Furthermore, the cyanobacteria-derived CQDs had almost no photobleaching under long-time UV irradiation, and exhibited high photostability in the solutions with a wide range of pH and salinity. Since no chemical reagent was involved in the synthesis of CQDs, the as-prepared CQDs were confirmed to have low cytotoxicity for PC12 cells even at a high concentration. Additionally, the CQDs could be efficiently taken up by cells to illuminate the whole cell and create a clear distinction between cytoplasm and nucleus. The combined advantages of green synthesis, cost-effectiveness and low cytotoxicity make synthesized CQDs a significant carbon source and broaden the application of cyanobacteria and provide an economical route to fabricate CQDs on a large scale.

## 1. Introduction

Carbon quantum dots (CQDs) have attracted considerable attention for their unique and tunable photoluminescence properties [1,2,3], easy of functionalization [4,5], high photostability [6], excellent biocompatibility [7,8,9] and negligible environmental impact [10]. Benefiting from these features and others, CQDs have been widely applied in electrochemical immune-sensing [11], bio-imaging [12,13,14], fluorescent probes [15], photocatalysis [16] and optoelectronics [17]. Over the past several years, extensive research efforts have focused on synthesizing CQDs with diverse emission performances and controlled chemical compositions using a variety of synthetic routes, such as arc discharge treatment [18], electrochemical exfoliation [19], laser ablation [20] and chemical oxidation [21]. Although recent progress has promoted the development of CQDs, most of these approaches usually suffer from the high cost of raw materials, complex procedures, harsh synthetic conditions or reliance on energy-consuming devices, which seriously limit the availability of large-scale production of the CQDs for practical applications. Among the proposed synthetic strategies for CQD fabrication, the hydrothermal method has been widely adopted as an effective bottom-up synthesis route for the preparation of CQDs from numerous molecules and has several distinct advantages, including simple preparation, low-cost equipment and economical consumption [22,23,24,25].

Over the past few years, the preparation of nanomaterials using renewable natural resources, environmentally friendly solvents and nontoxic chemicals has become the key issue that should be taken into consideration in a green synthetic strategy. Thus, enormous efforts have been put into developing green methods for synthesizing CQDs using natural biomass materials, such as grass [26], watermelon peels [27], banana juice [28] and strawberry juice [29]. Apart from flexible synthesis approaches, the preparation of biomass-based CQDs opens a new avenue for the value-added and sustainable utilization of useless materials. Motivated by this issue, it is still highly important and desirable to explore new carbon sources for the simple, economical, and green synthesis of CQDs. Cyanobacteria are phototrophic bacteria that exist in many eutrophic lakes, ponds and rivers throughout the world. Generally, heavily polluted water exposed to high temperatures and intense sunlight will provide optimum conditions for the fast growth of cyanobacteria, thus leading to a striking green color in the water [30]. As a renewable and low-cost biomass, cyanobacteria contains a large amount of nutrients and other available substances, which makes it attractive to convert this biomass to value-added products, such as organic fertilizer [31], high purity phycocyanin [32], activated carbon [33] and algae powder [34]. However, there are few reports regarding the use of cyanobacteria as a carbon source for the synthesis of fluorescent CQDs. As such, the exploration of cyanobacteria biomass as precursor for fluorescent nanomaterials is expected to improve the value of cyanobacteria and afford a new way for the low-cost fabrication of CQDs.

In this work, a simple, low-cost, and green synthesis strategy toward water-soluble CQDs via the hydrothermal heating of cyanobacteria as the sole carbon source was developed. Since cyanobacteria are rich in C, H, N and S elements [35,36], carbonization, surface functionalization and nitrogen doping occur simultaneously during the hydrothermal treatment, thus resulting in the formation of heteroatom-doped CQDs. Moreover, these resultant nanostructures with excellent fluorescence and low toxicity are demonstrated to be effective fluorescent probes for PC12 cell imaging.

## 2. Materials and Methods

### 2.1. Materials

Cyanobacteria powder was offered by Wuxi Delinhai Environmental Protection Technology Co., Ltd. (Wuxi, China). Purified water used here was purified through a Millipore system and used throughout the whole experiment. Quinine sulfate (99.0%) was purchased from Aladdin Biochemical Co., Ltd. (Shanghai, China). Phosphate buffer solutions (PBS) of different pH values were obtained from Haibiao Technologies Co., Ltd. (Fuzhou, China), All other reagents were purchased from Aladdin (Shanghai, China). Rat pheochromocytoma (PC12) were purchased from American Type Culture Collection (ATCC) (Manassas, VA, USA). CellTiter 96 AQueous One Solution Cell Proliferation Assay kit was from Promega (Madison, WI, USA).

### 2.2. Synthesis of CQDs

Cyanobacteria powder was first dried at about 60 °C for 48 h, until the water content decreased to less than 20%. Next, the dried sample was passed through a 60 mesh stainless steel sieve, and 0.2 g of pretreated cyanobacteria were dispersed into 20 mL of purified water under stirring. The mixture was transferred into a polytetrafluoroethylene-equipped stainless steel autoclave (50 mL) and heated at 180 °C for 12 h. After the reaction was complete and the reactant cooled to room temperature, a dark brown product was obtained, implying the formation of cyanobacteria-derived CQDs. The resulting mixture was filtered by a piece of microporous membrane with pore size of 0.22 μm to remove large particles, then dialyzed with a dialysis bag (MWCO ~ 800 Da) for 36 h to remove the reaction residues and byproducts. Finally, the solid cyanobacteria-derived CQDs were obtained by vacuum freeze-drying.

### 2.3. Characterization

The size and morphology of CQDs were investigated using a JEM-2100F microscope (JEOL, Akishima-shi, Tokyo, Japan) operated at an accelerating voltage of 200 kV. Raman spectrum with a 780 nm excitation laser were collected using a DXR780 Raman spectrometer (Thermo Scientific, New York, NY, USA). Fourier-transform infrared (FTIR) spectra were recorded on a VERTEX 80 V spectrometer in the range of 4000–400 cm^−1^ using the KBr pellet technique (Bruker, karlsruhe, Germany). X-ray photoelectron spectra (XPS) measurements were conducted on an AXIS UltraDLD spectrometer (Shimadzu, Kyoto, Japan). Elemental analysis was carried out on an Elementar Analysensysteme 2400 II (Perkin Elmer, Waltham, MA, USA). Ultraviolet-visible (UV-Vis) absorption spectrum was measured using a Lambda 950 UV-Vis spectrophotometer (Perkin Elmer, USA). Photoluminescent (PL) spectra and PL intensity were recorded using an F-7000 instrument (Hitachi, Tokyo, Japan), with the band pass for excitation and emission set as 10 nm. Time resolved spectra of CQDs were detected on a FLS 920 time-correlated single-photo-counting technique (EI, Edinburgh, UK) under an excitation of 410 nm.

The obtained lifetime value was determined via a double-exponential function *Y*(t) (Equation (1)) and the average lifetime was calculated according to Equation (2) [24].

(1) $$Y_{\left(t\right)}{\;=\;A}_{1}\exp\left(-t/\tau_{1}\right){\;+\;A}_{2}\exp\left(-t/\tau_{2}\right),$$

(2) $$\tau^{*}\;=\;\left(A_{1}\tau_{1}{}^{2}{\;+\;A}_{2}\tau_{2}{}^{2}\right)/\left(A_{1}\tau_{1\;}{+\;A}_{2}\tau_{2}\right),$$

where *A*_1_ and *A*_2_ refer to the weight of time-resolved fluorescence decay lifetimes *τ*_1_ and *τ*_2_, respectively, and τ* represents the average lifetime.

### 2.4. Quantum Yield Calculation

The quantum yield (QY) of the as-prepared CQDs was obtained according to an established relative method. Quinine sulfate was dissolved in 0.1 M H_2_SO_4_ (Φ = 54%) as a standard [37]. Both the absorbance of CQDs and quinine sulfate solutions were adjusted to below 0.1 to minimize inner filter effect. The QY of CQDs was determined by the following Equation:

(3) $${QY}_{x}{\;=\;QY}_{\mathrm{st}}\left(\frac{I_{x}}{I_{\mathrm{st}}}\right)\left(\frac{A_{\mathrm{st}}}{A_{x}}\right){\left(\frac{\eta_{x}}{\eta_{\mathrm{st}}}\right)}^{2},$$

where *I* and *A* are the fluorescence integral intensity and absorbance, respectively. The refractive index (*η*) of the solvent was 1.33. The subscripts x and st correspond to CQDs and quinine sulfate.

### 2.5. Cytotoxicity Assay

The cell viability was measured using an MTS assay. PC12 cells were first seeded in 96-well plates incubated at 37 °C in a 5% CO_2_ atmosphere, then incubated with CQDs at a concentration range from 0 to 1000 µg/mL. Each concentration was tested in 5 wells. The viability of the cells, after incubation with MTS reagent (one solution reagent) (20 μL/well) for 4 h, was determined by measuring the optical density (OD) at 490 nm using a SynergyTM 2 Multi-Function Microplate Reader (Bio-Tek Instruments, Inc. Winooski, VT, USA). The cell viability was estimated according to the following equation [13,38]:

(4) $$\mathrm{Cell}\;\mathrm{viability}\;\left(\%\right)=\frac{{OD}_{\mathrm{treated}}}{{OD}_{\mathrm{control}}}\;\times \;100\%,$$

where *OD*_control_ is the OD in the absence of CQDs and *OD*t_reated_ is the OD in the presence of CQDs.

### 2.6. Microscopic Imaging

PC12 cells were plated with 1 × 10^5^ cells/dishes in a glass-bottom plate and cultured for 24 h (37 °C, 5% CO_2_). Next, the cells were washed with PBS buffer. The diluted CQDs solution with a concentration of 500 μg/mL was introduced into the wells and further incubated for 24 h. After that, the excess CQDs were removed by washing three times with PBS buffer solution (pH = 7.4), and images were obtained using a confocal laser scanning microscope (LSM 700, Carl Zeiss, Jena, Germany) at 405 nm excitation.

## 3. Results and Discussion

### 3.1. Synthesis Process and Mechanism of CQDs

**Figure 1:**
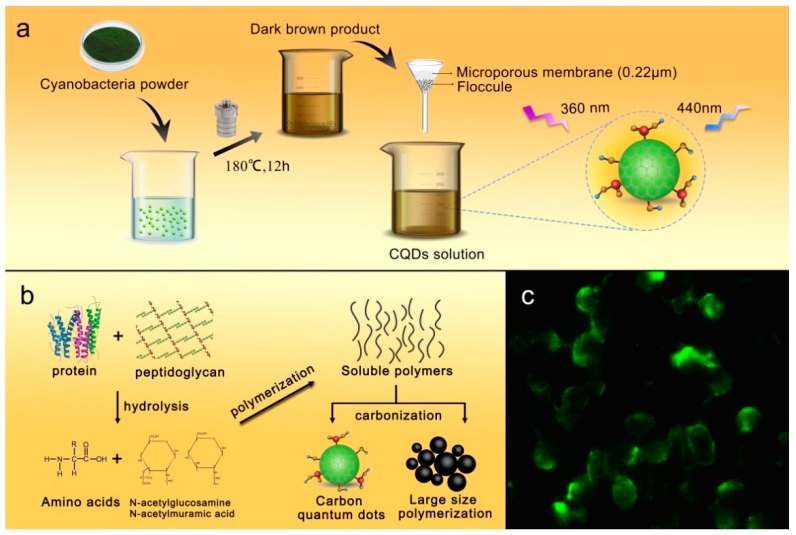
(**a**) Schematic route of CQDs fabricated from cyanobacteria; (**b**) a possible synthesis process of the cyanobacteria-derived CQDs; (**c**) fluorescence image of PC12 cells incubated with CQDs.

The synthesis route of the cyanobacteria-derived CQDs was schematically illustrated in Figure 1a. Firstly, the pretreated cyanobacteria powder was dispersed into purified water under stirring. Next, the mixture was transferred into a polytetrafluoroethylene-equipped stainless steel autoclave and heated at 180 °C for 12 h. After the reaction was complete and the reactant cooled down to room temperature, a dark brown product was obtained, implying the formation of cyanobacteria-derived CQDs. Afterwards, the resulting mixture was filtered by a piece of microporous membrane to remove large particles.

Based on the chemical composition of cyanobacteria and the mentioned analyses, we proposed a possible synthesis mechanism for the CQDs (Figure 1b). Commonly, the cyanobacteria are single-celled organisms that mainly contain proteins and peptidoglycan, and the elemental analysis revealed that the cyanobacteria were composed of elemental C (37.87%), H (8.04%), N (7.62%) and S (0.97%). It is reasonable to deduce that the cyanobacteria first suffered from hydrolysis under thermal conditions and produced large amounts of amino acid [39], *N*-acetylglucosamine acid and *N*-acetylmuramic acid [40]. Polymerization occurred during the synthetic process, and the formed soluble polymers were subjected to carbonization, thus facilitating the formation and growth of carbon cores [41]. Further, since complex compounds took part in the synthetic reaction, the surfaces of the CQDs were more likely to attach multiple functional groups after the reaction completed. These cyanobacteria-derived CQDs were used as fluorescent probes for biological imaging as shown in Figure 1c.

### 3.2. Chemical Structure of the CQDs

**Figure 2:**
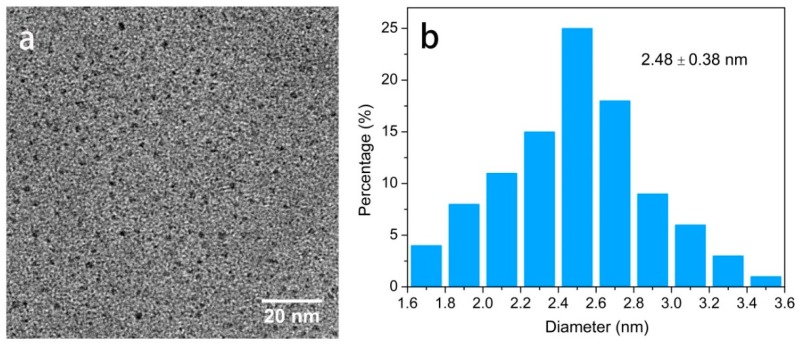
(**a**) HR-TEM image of the CQDs; (**b**) the size distribution of particles.

**Figure 3:**
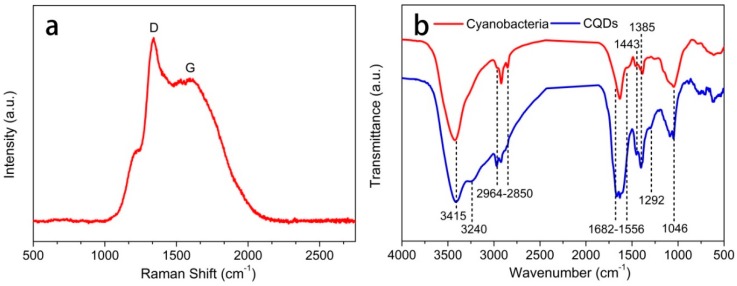
(**a**) Raman spectrum of the CQDs; (**b**) FTIR spectra of CQDs and cyanobacteria.

TEM characterization were performed to provide evidence of our successful synthesis of CQDs from the cyanobacteria. As shown in Figure 2a, the as-prepared CQDs exhibited almost spherical morphology and were well dispersed across the whole section without noticeable aggregation at room temperature. Furthermore, the corresponding particle-size histogram (Figure 2b) could be acquired through statistical analysis of around 100 nanoparticles, and the statistical results demonstrated that the size of CQDs ranged from 1.6 to 3.6 nm, with an average diameter of 2.48 nm. As a result, the narrow size distribution and the smaller average diameter were probably related to the uniform energy and high pressure that generated from the hydrothermal heating. Raman spectroscopy was used to analyze the defects in the CQDs. As shown in Figure 3a, two predominant peaks can be observed at 1340 cm^−1^ and 1590 cm^−1^, which are commonly attributed to the disordered D and crystalline G bands, respectively [42]. The intensity ratio of the D-band and G-band (I_D_/I_G_) of the CQDs was about 1.3, indicating a large amount of amorphous carbon within the structure of synthesized CQDs.

The surface functional groups of cyanobacteria and the as-prepared CQDs were characterized by FTIR spectra (Figure 3b). The wide absorption peak at 3415 cm^−1^ is attributed to intermolecular hydrogen bond O–H stretching vibrations and N–H stretching vibrations for both cyanobacteria and CQDs. The absorption band in the region of 2964–2850 cm^−1^ is related to the C–H stretching vibrations, and the absorption peaks located at 1443 cm^−1^, 1385 cm^−1^ and 1046 cm^−1^ are assigned to the stretching vibrations of C–H, C–N and C–O in the cyanobacteria and CQDs [43]. Notably, the N-containing groups not only existed in the chemical structure of cyanobacteria but also attached to the surface of the CQDs. The FTIR spectra of CQDs exhibited several peaks that were different from those of the cyanobacteria. Specifically, the absorption peaks at 1682–1556 cm^−1^ are ascribed to C=C and C=O bond stretching. The dull and intense absorption band is likely related to the increasing amount of C=C, which existed in the skeleton of the CQDs. One possible reason for this result is that thermal treatment tends to facilitate the precursor experiences of dehydration, decomposition, polymerization and carbonization, and thus leads to the formation of numerous carbon skeletons. The absorption peaks located at 3240 cm^−1^ and 1292 cm^−1^ are related to the stretching vibrations of –NH_2_ and the bending vibration of C–N for the cyanobacteria-derived CQDs. All of the above results demonstrate the presence of hydroxyl and carboxylic groups on the CQDs surfaces.

**Figure 4:**
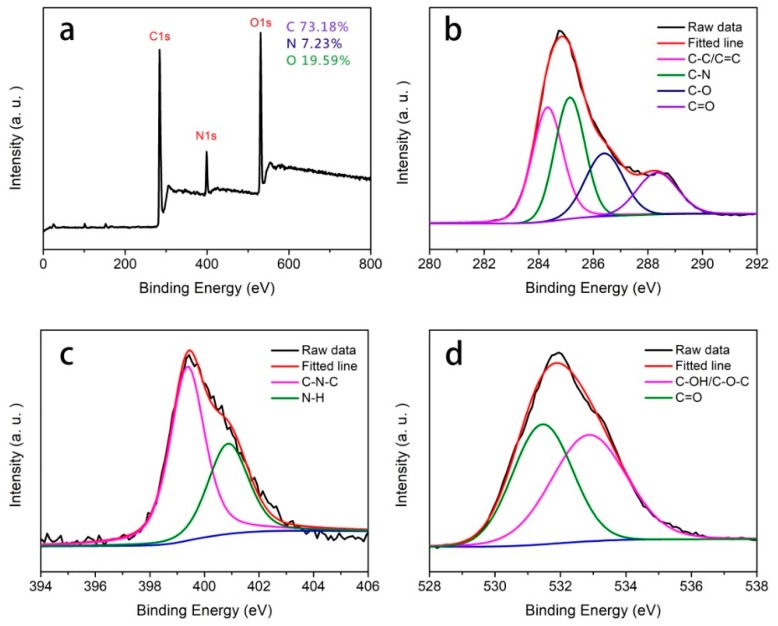
XPS full-survey spectrum of CQDs (**a**); high-resolution XPS of the C1s (**b**), N1s (**c**) and O1s (**d**) spectra.

The chemical structure and composition of CQDs were further identified by XPS spectra. As shown in Figure 4a, the XPS survey spectra of the obtained CQDs displayed three typical strong peaks at 284.7, 399.4 and 531.9 eV, which were attributed to C1s, N1s, and O1s, respectively, indicating that the CQDs mainly contained C (73.18%), N (7.23%) and O (19.59%) elements. In the C1s spectra (Figure 4b), four peaks appeared at 284.3, 285.1, 286.4 and 288.4 eV originating from the sp^2^ C–C/C=C, C–N, C–O, C=O groups, respectively [44]. The N1s band of CQDs (Figure 4c) can be deconvoluted into two peaks at 399.4 and 400.9 eV, representing pyrrolic N and amine groups, respectively. The pyrrolic N formed by the dehydrolysis reaction between carboxyl and amine groups was the main component of N in the as-prepared CQDs. Previous reports have proposed that the pyrrolic N is more likely to increase the electronic cloud density of CQD surfaces, and thus result in an enhancement of luminescence efficiency [24,26]. In the O1s spectra (Figure 4d), the peaks at 531.5 and 532.9 eV are assigned to the binding energies of C=O and C–O, respectively [45]. Overall, the resultant CQDs contained multiple O- and N-related functional groups that were not only responsible for their excellent water solubility, but also could be highly related to their fluorescence emission.

### 3.3. Optical Performance of the CQDs

**Figure 5:**
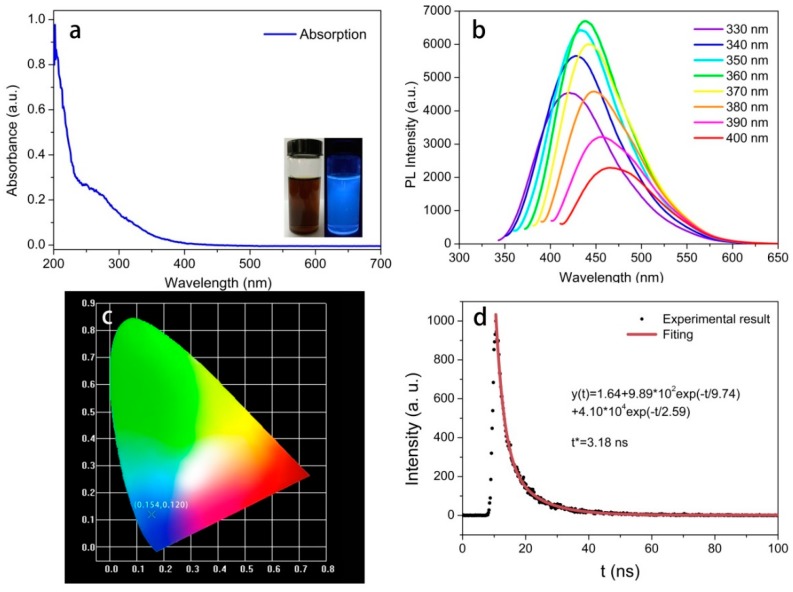
(**a**) Absorption spectrum of the CQDs under 360 nm excitation; inset: optical images under daylight (left) and UV light (right); (**b**) PL emission spectra of the CQDs at increasing excitation wavelengths from 330 to 400 nm in 10-nm increments; (**c**) CIE chromaticity diagram of the CQDs; (**d**) fluorescence decay curve of CQDs fitted by two-order exponential functions, and the fitting Equation is shown in the corresponding panel.

The optical performance of the as-prepared CQDs was characterized by UV-Vis absorption spectroscopy and PL measurements. The prepared aqueous CQD solution displayed a brown color under visible light, while the diluted CQD solution exhibited strong blue fluorescence under UV irradiation (365 nm) (inset in Figure 5a). As shown in Figure 5a, the optical absorption peak of the CQDs was observed in the ultraviolet region, with a maximum absorption at nearly 270 nm, corresponding to the n-π* transition of the C=O within the structure of the CQDs [46]. The PL spectra in Figure 5b show that the maximum PL intensity can be obtained under 360 nm excitation, and the optimal emission wavelength was determined to be 439 nm, which further demonstrates blue fluorescence of cyanobacteria-derived CQDs. Significantly, as the excitation wavelength increased from 330 to 360 nm, corresponding fluorescence intensity gradually increased up to a maximum value of 360 nm excitation before decreasing as the excitation wavelength increased 360–400 nm. These results suggest a typical excitation-dependent emission behavior for cyanobacteria-derived CQDs. Such optical properties associated with surface states have been illustrated in many previous reports [47,48,49], and the surface-contained functional groups tend to introduce various energy levels, which result in a series of emissive traps on the surface of CQDs. Commonly, the surface states are not introduced by a single chemical group, but the hybridization of the carbon backbone and connected chemical groups. The certain excitation wavelength is beneficial to the activation of some emissive trap sites, thus giving rise to a corresponding PL emission. It should be mentioned that a higher degree of surface oxidation or other effective modification can result in more surface defects, and thus the PL emission exhibited red-shifted emission behavior. The fluorescence QY of the CQDs was determined to be ~9.24%; this result was significantly better than the QY of other biomass-based CQDs in previous reports [26,27,28,29]. Moreover, when irradiated by 365 nm ultraviolet light, the chromaticity coordinates of CQDs were in the blue light region (x = 0.154, y = 0.120), which further confirmed the typical blue emission behavior (Figure 5c).

To gain insight into the optical performance, the fluorescence lifetime of CQDs was also investigated in Figure 5d. The average lifetime was determined to be 3.18 ns, according to Equations (1) and (2). Such a short lifetime reveals that the luminescence of the CQDs can be caused by the defect states on the surfaces of CQDs, and that these surface states have the capacity to result in the radiative recombination of electrons and holes and then release energy in the form of photo emissions [50]. Moreover, the lifetime of synthesized CQDs is composed of two proportions, involving a short-lived component τ_1_ and long-lived component τ_2_. Previous works have pointed out that the lifetime τ_1_ and τ_2_ were related to the core states and surface states of CQDs, respectively [51,52,53]. In our case, the weight of *τ*_1_ and *τ*_2_ were determined to be 8.32% and 91.68%, respectively, and this result demonstrates that the PL emission is mainly controlled by the surface states induced by multiple functional groups within the structure of the CQDs.

### 3.4. Stability of the CQDs

**Figure 6:**
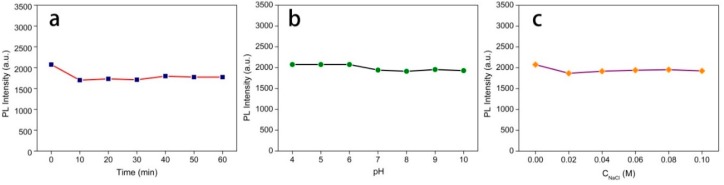
Effect of ultraviolet light time (**a**), pH (**b**) and NaCl concentration (**c**) on the PL intensities of the CQDs.

Due to the importance of fluorescence stability for CQDs, the effect of UV light irradiation, acidic/basic and ionic strength conditions of the PL intensity were investigated [54]. In Figure 6a, the synthesized CQDs were irradiated by UV light (365 nm) for 60 min, and the results illustrate that the PL intensity exhibited negligible variation along with the exposure time. In addition, no significant change was observed in PL intensity of CQDs, with the pH increasing from 4 to 10 (Figure 6b). The high stability of CQDs in the pH range of 4–10 made them very useful for bio-applications, as most biological activities happen in this pH range. With the increase of the pH value, the intensity of the emitted light remained almost unchanged in the acidic and neutral environment. Figure 6c illustrates that the PL intensity remained almost unchanged even as the NaCl concentration reached 0.1 M. These results were consistent with previous reports on the characteristics of carbon dots [14,55]. The above-mentioned analyses demonstrate that the as-prepared CQDs possess high stability, and such excellent fluorescence is conducive to biological imaging applications.

### 3.5. Biological Imaging

**Figure 7:**
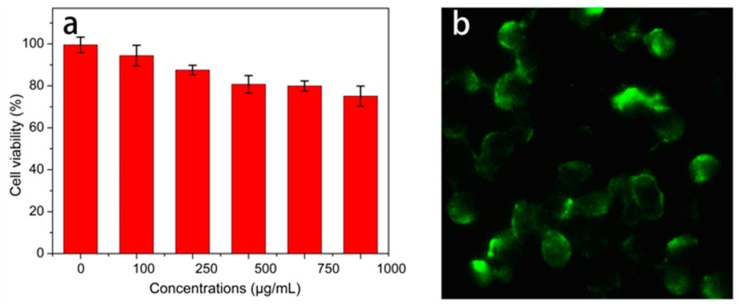
(**a**) Cell viability assays of the PC12 cells treated with different concentrations of CQDs for 24 h; (**b**) fluorescence image of PC12 cells incubated with CQDs (500 μg/mL) for 24 h under excitation wavelength of 405 nm.

Considering the cytotoxicity of CQDs was important for their potential biomedical applications [56,57], the cell viability values of PC12 cells were determined after cocultivation with CQD solution. As shown in Figure 7a, the percentage of survival cells remained at 94.4% when the CQD concentration was up to 100 μg/mL. Indeed, even at the higher concentration (500 μg/mL), the cell viability was still >80%. Under the same test conditions, the toxicity of CQDs in Raji cells were also carried out and the percentage of survival cells remained at more than 85% with 500 μg/mL CQDs. While CQDs were added to Raji cells, it was difficult to find the fluorescence markers under the fluorescence microscope. We speculated that the size of the Raji cells might have been too small to be observed under the microscope magnification in our laboratory. The above results imply that the cyanobacteria-derived CQDs exhibited excellent biocompatibility and low cytotoxicity. Considering their bright fluorescence, resistance to photobleaching, great water dispersibility and low cytotoxicity, these CQDs are promising candidates for biomedical applications.

As displayed in Figure 7b, the PC12 cells were labeled with the synthetic CQDs after incubation in a medium containing 500 μg/mL CQDs for 24 h. It should be noted that the fluorescence can hardly be observed at the center positions of the cells. These center areas were likely to be the locations of the cell nuclei. Thus, it can be speculated that these CQDs were easily taken up by the cells through endocytosis, and were located mainly at the cytoplasm. This result is consistent with those reported previously [12,13,14]. Therefore, a conclusion is drawn that these CQDs can be used as fluorescent probes for biological imaging and other related biomedical applications.

## 4. Conclusions

In summary, a one-step hydrothermal method was adopted to prepare CQDs from cyanobacteria at a constant temperature of 180 °C for 12 h for the first time. The synthesized cyanobacteria-derived CQDs were mono-dispersed with an average diameter of 2.48 nm and exhibited excitation-dependent PL emissions with a QY of 9.24%. Since no chemical reagent was involved in the synthesis of CQDs, the as-prepared CQDs are confirmed to have low cytotoxicity for PC12 cells even at a high concentration. Additionally, the CQDs were efficiently taken up by cells and wholly illuminated them, creating a clear distinction between cytoplasm and nucleus. The successful fabrication of CQDs from cyanobacteria, as well as their determined valuable application, not only provides an attractive solution to the pressing global clean water shortage problem, but also broadens the application of cyanobacteria and offers a significant large-scale commercial method for the preparation of CQDs.
